# Specialist nurses’ perceptions of the barriers and facilitators to inviting adult NHS patients to participate in clinical research studies: a qualitative descriptive study

**DOI:** 10.1186/1745-6215-16-S2-P94

**Published:** 2015-11-16

**Authors:** Caroline French, Charitini Stavropoulou

**Affiliations:** 1 City University, London, UK; 2 Solent NHS Trust, Southampton, UK

## Background

Specialist nurses often collaborate with researchers to facilitate recruitment of adult NHS patients, yet little is known about their experiences of inviting participation to research studies. It is well documented that clinical staff involved with recruitment often do not invite all potentially eligible patients, leading to under-recruitment and selection bias. Many barriers and facilitators perceived by clinical staff to inviting research participation have been identified, the majority of these relating to physicians or specific randomised controlled trials.

## Aims

This study aims to explore the barriers and facilitators perceived by specialist nurses to inviting adult patients to a variety of research studies in different NHS settings.

## Methods

The study employs a cross-sectional qualitative descriptive design using purposive and convenience sampling, semi-structured individual interviews and Framework thematic analysis. This study forms an MRes student dissertation and will be completed in September 2015. To date 12 specialist nurses have been recruited, representing a variety of clinical specialties and employing NHS Trusts, with experience of inviting patients to a broad variety of research studies. Data collection is ongoing and 6 interviews have been conducted thus far.

## Results

Emerging preliminary themes include the perceived facilitator of rapport and clinical credibility within the nurse / patient relationship, and the perceived barrier of insufficient understanding of individual research studies by the nurse.

## Conclusions and implications

Increased understanding of both barriers and facilitators may inform development of evidence-based interventions to optimise research recruitment involving specialist nurses and other healthcare staff.

